# Evans Syndrome in Children: Long-Term Outcome in a Prospective French National Observational Cohort

**DOI:** 10.3389/fped.2015.00079

**Published:** 2015-09-29

**Authors:** Nathalie Aladjidi, Helder Fernandes, Thierry Leblanc, Amélie Vareliette, Frédéric Rieux-Laucat, Yves Bertrand, Hervé Chambost, Marlène Pasquet, Françoise Mazingue, Corinne Guitton, Isabelle Pellier, Françoise Roqueplan-Bellmann, Corinne Armari-Alla, Caroline Thomas, Aude Marie-Cardine, Odile Lejars, Fanny Fouyssac, Sophie Bayart, Patrick Lutz, Christophe Piguet, Eric Jeziorski, Pierre Rohrlich, Philippe Lemoine, Damien Bodet, Catherine Paillard, Gérard Couillault, Frédéric Millot, Alain Fischer, Yves Pérel, Guy Leverger

**Affiliations:** ^1^Department of Pediatric Hematology, University Hospital of Bordeaux, Bordeaux, France; ^2^Centre de Référence National des Cytopénies Autoimmunes de l’Enfant (CEREVANCE), University Hospital of Bordeaux, Bordeaux, France; ^3^CIC 0005, INSERM CICP, University Hospital of Bordeaux, Bordeaux, France; ^4^Department of Hematology, APHP – Hôpital Robert Debré, Paris, France; ^5^Centre de Référence National des Cytopénies Autoimmunes de l’Enfant (CEREVANCE), APHP – Hôpital Robert Debré, Paris, France; ^6^Immunogenetics of Pediatric Autoimmune Diseases, Institut Imagine, INSERM UMR_S1163, Université Paris Descartes, Paris, France; ^7^Pediatric Immuno-Hematology Unit, University Hospital of Lyon – IHOP, Lyon, France; ^8^Department of Pediatric Hematology, University Hospital Timone Enfants, Marseille, France; ^9^Hôpital des Enfants, University Hospital of Toulouse, Toulouse, France; ^10^Department of Pediatrics, Hôpital Jeanne de Flandre, University Hospital of Lille, Lille, France; ^11^Department of Pediatrics, APHP – Hôpital Bicêtre, Le Kremlin-Bicêtre, France; ^12^Pediatric Hemato-Oncology Unit, University Hospital of Angers, Angers, France; ^13^Pediatric Hemato-Oncology Unit, University Hospital of Nice, Nice, France; ^14^Department of Pediatrics, University Hospital of Grenoble, Grenoble, France; ^15^Pediatric Hemato-Oncology Unit, Hôpital Mère Enfant, University Hospital of Nantes, Nantes, France; ^16^Pediatric Immuno-Hematology-Oncology Unit, University Hospital of Rouen, Rouen, France; ^17^Pediatric Hemato-Oncology Unit, Centre de Pédiatrie Gatien De Clocheville, University Hospital of Tours, Tours, France; ^18^Service de Médecine Infantile II, Hôpital d’Enfants, University Hospital of Nancy, Vandoeuvre-lès-Nancy, France; ^19^Department of Pediatric Hematology, Hôpital Sud, University Hospital of Rennes, Rennes, France; ^20^Pediatric Hemato-Oncology Unit, Hôpital de Hautepierre, University Hospital of Strasbourg, Strasbourg, France; ^21^Pediatric Hemato-Oncology Unit, Hôpital Mère Enfants, University Hospital of Limoges, Limoges, France; ^22^Department of Pediatric Hematology, Hôpital Arnaud de Villeneuve, University Hospital of Montpellier, Montpellier, France; ^23^Centre de Référence National des Cytopénies Autoimmunes de l’Enfant (CEREVANCE), Hôpital Arnaud de Villeneuve, University Hospital of Montpellier, Montpellier, France; ^24^Pediatric Hemato-Oncology Unit 1, University Hospital of Besançon, Besançon, France; ^25^Onco-Hematology Unit, Hôpital Morvan, University Hospital of Brest, Brest, France; ^26^Onco-Hematology Unit, University Hospital of Caen, Caen, France; ^27^Pediatric Hemato-Oncology Unit, Hôtel-Dieu, University Hospital of Clermont-Ferrand, Clermont-Ferrand, France; ^28^Pediatric Hemato-Oncology Unit, Hôpital d’Enfants, University Hospital of Dijon, Dijon, France; ^29^Pediatric Hemato-Oncology Unit, University Hospital of Poitiers, Poitiers, France; ^30^Department of Immuno-Hematology, APHP – Hôpital Necker-Enfants Malades, Paris, France; ^31^Centre de Référence National des Cytopénies Autoimmunes de l’Enfant (CEREVANCE), APHP – Hôpital Necker-Enfants Malades, Paris, France; ^32^Department of Onco-Hematologie, APHP – Hôpital Trousseau, Paris, France; ^33^Centre de Référence National des Cytopénies Autoimmunes de l’Enfant (CEREVANCE), APHP – Hôpital Trousseau, Paris, France

**Keywords:** immune thrombocytopenic purpura, autoimmune hemolytic anemia, child, Evans syndrome

## Abstract

Evans syndrome (ES) is a rare autoimmune disorder whose long-term outcome is not well known. In France, a collaborative pediatric network set up via the National Rare Disease Plan now provides comprehensive clinical data in children with this disease. Patients aged less than 18 years at the initial presentation of autoimmune cytopenia have been prospectively included into a national observational cohort since 2004. The definition of ES was restricted to the simultaneous or sequential association of autoimmune hemolytic anemia (AIHA) and immune thrombocytopenic purpura (ITP). Cases were deemed secondary if associated with a primitive immunodeficiency or systemic lupus erythematosus. In December 2014, we analyzed the data pertaining to 156 children from 26 centers with ES whose diagnosis was made between 1981 and 2014. Median age (range) at the onset of cytopenia was 5.4 years (0.2–17.2). In 85 sequential cases, the time lapse between the first episodes of AIHA and ITP was 2.4 years (0.1–16.3). The follow-up period as from ES diagnosis was 6.5 years (0.1–28.8). ES was secondary, revealing another underlying disease, in 10% of cases; various associated immune manifestations (mainly lymphoproliferation, other autoimmune diseases, and hypogammaglobulinemia) were observed in 60% of cases; and ES remained primary in 30% of cases. Five-year ITP and AIHA relapse-free survival were 25 and 61%, respectively. Overall, 69% of children required one or more second-line immune treatments, and 15 patients (10%) died at the age of 14.3 years (1.7–28.1). To our knowledge, this is the first consistent long-term clinical description of this rare syndrome. It underscores the high rate of associated immune manifestations and the burden of long-term complications and treatment toxicity. Future challenges include (1) the identification of the underlying genetic defects inducing immune dysregulation and (2) the need to better characterize patient subgroups and second-line treatment strategies.

## Introduction

The association of immune thrombocytopenic purpura (ITP) and autoimmune hemolytic anemia (AIHA), both mediated by autoantibodies and responding to splenectomy, was first described in 1951 in 24 patients aged 3–78 years by Robert Evans (1). Some cases of Evans syndrome (ES) were associated, mainly in adults, with various medical conditions such as systemic lupus erythematosus (SLE) or malignancies (2). In children, autoimmune cytopenia may indicate the presence of an underlying primitive immunodeficiency (PID) (3). ES has a chronic and relapsing course, and patients are usually dependent on prolonged immunosuppressive treatments. Specific effective and non-toxic therapies are still elusive (4).

Although the incidence of childhood ITP has been estimated at two to five cases per 100,000 per year in children less than 18 years (5), the precise incidence of childhood AIHA and ES has not yet been determined. The literature on pediatric ES is composed of isolated case reports and limited retrospective or questionnaire-based series (Table 1) (6–9). In the last 30 years, less than 100 heterogeneous cases of ES based on a variety of definitions have been published, and consistent clinical data regarding underlying diseases and long-term follow-up are still lacking.

**Table 1:** **Main series of ES children**.

Reference	Type of study	Number of centers	Number of patients	Period of study years	Median follow-up years	Number of “primary^a^” ES
(7)	Retrospective	1	11	18	4.8	7
(9)	Prospective	1	10	11	7	2
(6)	Retrospective	21	42	24	3	?
(8)	Prospective	1	11	18	8	11
Present study (2015)	Prospective	26	156	34	6.8	47

^a^*Various definitions, follow-up modalities, and durations and data collection*.

A national network for autoimmune cytopenia in children was created in France in 2004, within the scope of a Rare Disease Plan further developed in 2007. The network’s working model is similar to oncology networks, including referral pediatric and adult hematological centers in each region. A retrospective pilot study conducted during the 1990–2002 period accreted clinical, biological, and treatment data in 36 children with ES (10). Since 2004, a formal national prospective observational cohort of children with AIHA, chronic ITP, and ES known as OBS’CEREVANCE has been set up (11).

The aim of the present collaborative work is to present comprehensive long-term follow-up clinical data regarding a large national cohort of non-selected children with ES.

## Patients and Methods

### Patient selection and data collection

Since 2004, French patients aged less than 18 years presenting with autoimmune cytopenia have been consecutively included into the national prospective observational OBS’CEREVANCE cohort. This study was approved by Bordeaux’s Institutional Review Board on Human Research. Written informed consent was obtained from the elder patients and/or the subjects’ parents and/or the persons with parental authority.

The pediatric hematologists and their referring physicians from general hospitals or adult teams prospectively forwarded in real time all consultation or hospitalization records for each visit to the coordinating center. For each patient, relevant clinical, biological, and treatment data from birth to last follow-up were prospectively collected, coded, and integrated by clinical research assistants into the OBS’CEREVANCE Access database (Microsoft, Redmond, WA, USA). This database was declared to the French commission for information technology and civil liberties (CNIL November 9, 2009, 1396823V0). For the present study, the database was analyzed on December 31, 2014.

### Inclusion criteria

Patients with ES defined as the simultaneous (within 1 month) or sequential association of ITP and AIHA were included in the OBS’CEREVACE cohort. ITP was defined according to the international working group criteria, i.e., a platelet count <100 G/L on two separate occasions (12). AIHA was defined by an Hb level <11 g/dL with a positive direct antiglobulin test (DAT) and at least one hemolysis criteria among the following: reticulocytosis >120 g/L, free bilirubin >17 μmol/L, and haptoglobin <10 mg/dL.

### Exclusion criteria

Patients with isolated DAT and/or compensated hemolysis without anemia or patients with antiplatelet antibodies without thrombocytopenia were not considered to have ES. Patients with inherited red blood cell or platelet diseases, with autoimmune cytopenia associated with chemotherapy, bone marrow transplantation, solid organ transplantation, or human immunodeficiency virus, and patients not living in metropolitan France were not included in this descriptive study.

### Patient management

The existence of consanguinity or immune manifestations in first-degree relatives (father, mother, or siblings) and the complete history of included children were recorded, with special attention to the initial presentation of AIHA and ITP.

Historically, French pediatric hematology centers have adopted common homogeneous practices. Etiological investigations upon initial diagnosis were consistent with national guidelines and were annually repeated during follow-up (http://www.cerevance.org). Extensive screening for infectious agents was conducted and documented. Blood smears were analyzed to assess platelet volume, red cell morphology, and presence of schistocytes. Complete B- and T-lymphocyte immunophenotyping was performed in national CEREDIH network laboratories. Functional and genetic immune-derived studies to identify known genetically identified PIDs were sent mainly to the French reference laboratory (CEDI, Dr. C. Picard and Prof. A. Fischer, Necker Hospital, Paris). In particular, most children underwent screening tests for autoimmune lymphoproliferative syndrome (ALPS): initial lymphoproliferation, hypergammaglobulinemia, or high counts of circulating TCR αβ+ CD4− CD8− double-negative T lymphocytes (DNTs) led to further investigations looking at plasma levels of interleukin (IL)-10 and Fas ligand (FasL), functional apoptosis tests, and gene sequencing (13). Clinical screening for SLE or other autoimmune diseases was conducted at each visit or as required, and usual biological or urinary tests were regularly performed.

In this observational real-life study, treatments and supportive care were administered according to the CEREVANCE group or other published guidelines (14–18). First-line therapies comprised mainly intravenous immunoglobulin (IVIg) or steroids in ITP and steroids only in AIHA. Second-line therapies, such as azathioprine, anti-D, cyclosporine, colchicine, hydroxychloroquine, mycophenolate mofetil, rituximab, splenectomy, thrombopoietin-receptor agonists, were given sequentially or in combination in the presence of steroid resistance or dependence, or in case of relapses.

### Definitions

#### Severity of AIHA and ITP at Initial Diagnosis

A severe clinical condition was defined in ITP as clinical severity score ≥3, as defined by the Buchanan’s score [overall bleeding severity was graded on a scale of 0 (none) to 5 (life-threatening or fatal), mucosal hemorrhages defined a grade of 3 (moderate)] (19), and in AIHA as AIHA CEREVANCE score ≥2 (grade 0: no sign of anemia; grade 1: pallor, fatigue, no impact on daily life; grade 2: tachycardia, malaise, dizziness, significant impact on daily life; grade 3: cardiac, renal, or neurological impairment). A severe hematological condition was defined in ITP as a platelet count <10 g/L and in AIHA as an Hb level <7 g/dL.

#### Neutropenia

Neutropenia, of presumed immune origin, with or without anti-neutrophil autoantibodies, was defined by peripheral neutropenia <1 g/L lasting more than 6 months, provided that an infectious or drug-induced cause was excluded.

#### Primary and Secondary ES

In primary ES cases, no immune manifestations could be identified during the entire follow-up. Secondary ES cases were associated with a primary genetically identified immunodeficiency (PID) or SLE according to validated criteria (20–22).

#### ITP and AIHA Outcome

At the last follow-up, the status of each cytopenia was as follows: no remission (NR): platelet count <30 g/L or Hb <7 g/dL; partial remission (PR): platelet count 30–100 g/L or Hb 7–11 g/dL with reticulocytes >120 g/L; complete remission (CR): platelet count >100 g/L and Hb ≥11 g/dL with reticulocytes ≤120 g/L, and ongoing treatment or treatment discontinued since less than 12 months; continuous complete remission (CCR): CR without any treatment for at least 12 months. A relapse of ITP or AIHA was defined by the recurrence of ITP and/or AIHA after PR or CR.

### Statistical analysis

All statistical analyses were performed using NCSS 2001 software (23). The distribution of qualitative variables between groups was compared with the chi-square test or with the Fisher’s exact test. ITP and AIHA relapse-free survival (RFS) was estimated using the Kaplan–Meier method.

## Results

### Patient characteristics

**Figure 1:**
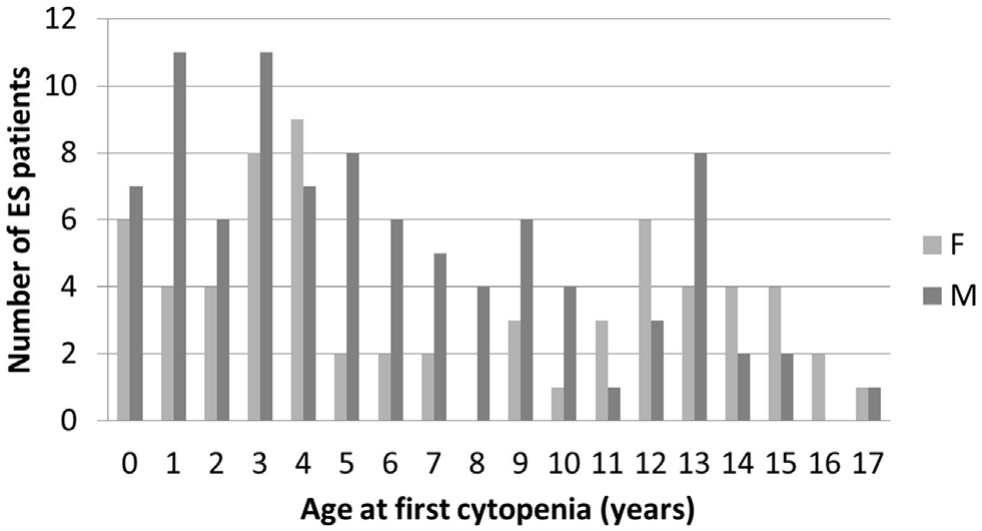
**Age and gender distribution at initial diagnosis of the first cytopenia in 156 children with ES**.

In December 2014, data from 156 children followed in 26 centers, diagnosed between July 1981 and January 2015 were analyzed. All families to whom the study was proposed consented to participate in the study. Median (range) age at initial cytopenia was 5.4 years (0.2–17.2). The sex ratio was 1.44 (92/64). There was a tendency for male predominance in younger children and female predominance in older children (Figure 1).

Eleven of 156 (4%) children were of consanguineous parents and 34/156 (22%) had one or more than one first-degree relative with immune manifestations, mainly AID, of whom 5 had presented with autoimmune cytopenia. At birth, 5/94 (5%) children were premature (<37 weeks gestational age) and in 9/84 (11%) birth weight was <10th percentile for gestational age.

Initial cytopenia was AIHA in 39 patients (25%), ITP in 46 patients (29%), and simultaneous AIHA and ITP in 71 patients (46%). When sequential, the median time lapse between the two cytopenias was 2.4 years (0.1–16.3). The clinical presentation was severe in 41/102 (40%) ITP patients (five score 4 patients) and in 65/111 (59%) AIHA patients (17 grade 3 patients). Hematological presentation was severe in 69/134 (51%) ITP patients and in 68/139 (49%) AIHA patients. DAT type was consistently IgG or IgG plus complement. An infection was identified upon initial diagnosis of ES in 5 simultaneous ITP/AIHA cases (EBV, *n* = 3; one tuberculosis, *n* = 1; parvovirus, *n* = 1) and in 13 sequential cases (CMV, *n* = 4; parvoviruses, *n* = 3; varicella-zoster, *n* = 2; mycoplasma, *n* = 2; EBV, *n* = 1; and rotavirus, *n* = 1).

In 32 children (20%), associated immune neutropenia was present, prior to the onset of ES in two patients and during or after the diagnosis of ES in 30 patients. Specific autoantibodies were identified in 67% of the 21 patients tested. Clinical consequences were mouth ulcers or diarrhea in 33% of cases, one or more episodes of severe WHO grade 3–4 bacterial sepsis in 43%. Hematopoietic growth factor was required in 14% of cases.

### Diagnosis

Autoimmune lymphoproliferative syndrome–FAS was identified in three children upon initial diagnosis of ES (two simultaneous, one sequential). They were 4, 11.8, and 13.5 years old at that time and had had previous significant lymphoproliferation (supracentimetric cervical adenopathies or persistent enlarged spleen besides hemolysis). All met the international criteria for ALPS: elevated plasma levels of double-negative T cells, FasL, IL-10, and defective *in vitro* Fas-induced lymphocyte apoptosis (20). All three had germline mutations in the *TNFRSF6* gene.

Thirteen children were diagnosed with SLE. ES occurred at a median age of 14 (1–16). Main SLE clinical criteria were achieved concomitantly with the onset of ES in 3/13 patients and later within a 1- to 10-year period in 10/13 patients. Two patients had associated acquired thrombotic thrombocytopenic purpura.

None were diagnosed for malignancy. In 93 children (60%), various associated immune clinical or biological manifestations were noted, up to 6 years prior to and 15.4 years following ES diagnosis. This consisted mainly of significant lymphoproliferation, other autoimmune diseases, and hypogammaglobulinemia. Investigations of PID are being conducted in this subgroup.

Finally, ES was secondary in 10% (*n* = 16), primary in 30% (*n* = 47), and unclassified in 60% (*n* = 93) of patients.

### Management

**Figure 2:**
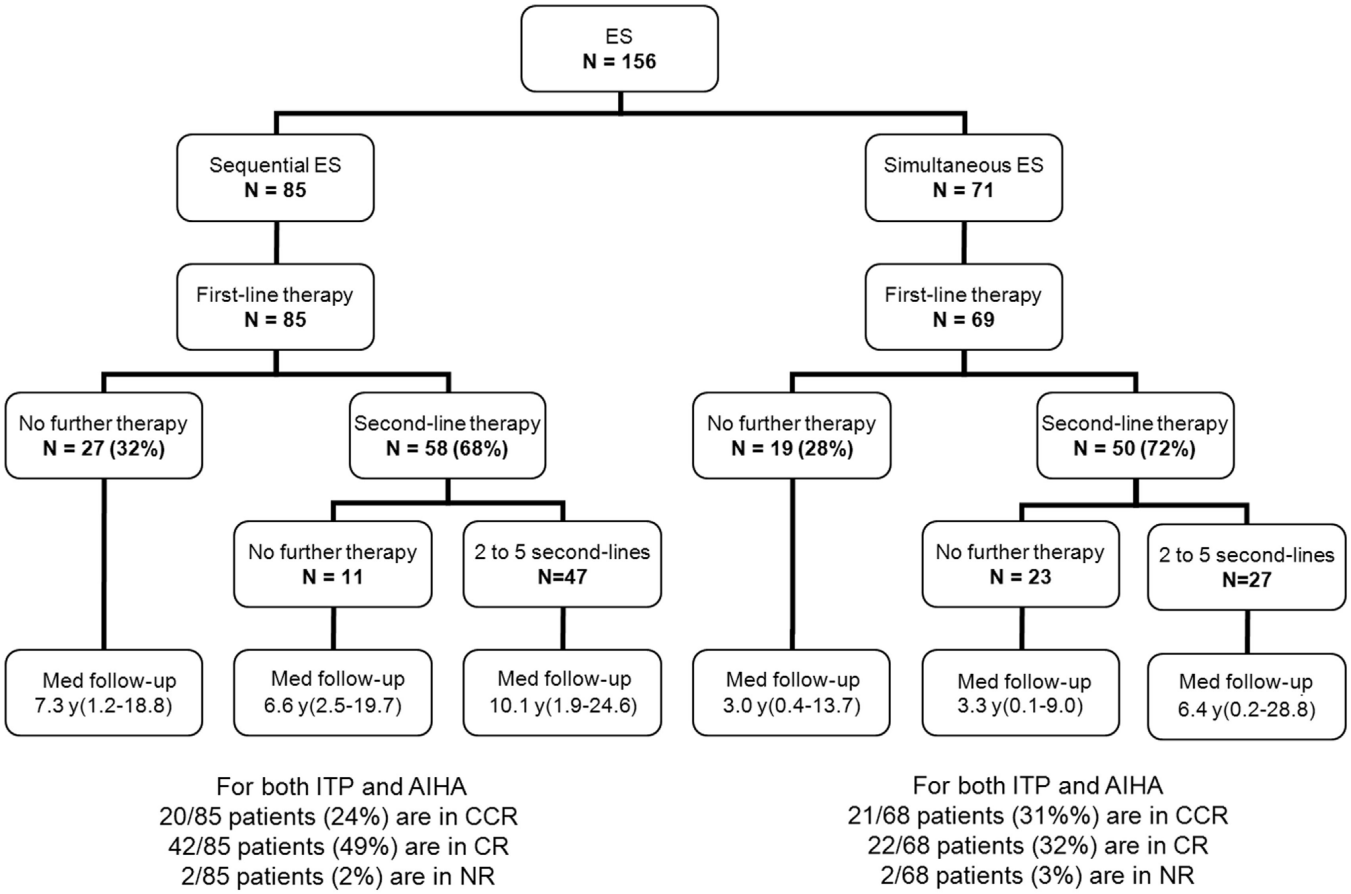
**Management and outcome of 156 children with ES**.

Besides first-line therapy with IVIg or steroids, 108 children (69%) required a variety of second-line treatments; 74/156 (47%) children received more than one second-line treatment (from two to seven). ITP was the main treatment target. The median time interval between initial diagnoses and the introduction of a second-line therapy was longer in sequential forms (1.8 years) than in simultaneous forms (0.4 years). This is due to prolonged “watch and wait” policy whenever isolated persistent or chronic ITP was the initial cytopenia (Figure 2). Secondary ES and unclassified cases significantly required second-line treatments compared to primary cases (Table 2).

**Table 2:** **Variables associated with the necessity of a second-line treatment**.

	Patients not requiring second-line therapy	Patients requiring second-line therapy
	*N* **=** 48 (31%)	*N* **=** 108 (69%)
Median age at initial cytopenia, years (min–max)	5.0 (0.3–17.2)	5.7 (0.2–17.0)
Median age at ES diagnosis, years (min–max)	8.9 (0.3–17.2)	8.6 (0.2–19.4)
Sex ratio	0.55 (17/31)	0.77 (47/61)
Prematurity, % (*n*)	3 (1/32)	6 (4/62)
Consanguinity, % (*n*)	5 (2/39)	10 (9/87)
First-degree relatives’ immune events, % (*n*)	20 (10/48)	22 (24/108)
Occurrence of ITP and AIHA		
Simultaneous, % (*n*)	44 (21/48)	46 (50/108)
Sequential, % (*n*)	56 (27/48)	54 (58/108)
– AIHA as initial manifestation, % (*n*)	27 (13/48)	24 (26/108)
– ITP as initial manifestation, % (*n*)	29 (14/48)	30 (32/108)
Associated neutropenia, % (*n*)	18 (9/48)	21 (23/108)
Primary ES, % (*n*)	45 (21/47)^*§^	55 (26/47)
Secondary ES, % (*n*)	6 (1/16)	94 (15/16)
Unclassified, % (*n*)	28 (26/93)	72 (67/93)

*p < 0.05, primary vs. secondary ES.

*^§^p < 0.05, primary vs. others*.

In this observational “real-life” study, the main second-line immunosuppressive therapy were rituximab (*n* = 34), azathioprine (*n* = 16), splenectomy (*n* = 13), cyclosporine (*n* = 10), and mycophenolate mofetil (*n* = 3). In sequential forms, various immunosuppressive and other treatments given for the initially isolated cytopenia, including splenectomy (*n* = 7), did not avoid the occurrence of the second episode, several months and up to 16 years later. The rank order of treatments administered in small heterogeneous subgroups is under current analyses.

### Outcome

Median follow-up time following ES diagnosis was 6.5 years (0.1–28.8). Overall, 15 patients (10%) died at a median age of 14.3 years (1.7–28.1). Disease-related causes accounted for 30% of deaths (four cases of cerebral or gastrointestinal hemorrhages, and one severe vasculitis in SLE); in 70% of deaths, all of infectious origin, the underlying disease and treatments were suspected to have played a role. Median age for the 141 surviving patients at last follow-up was 16.1 years (1.6–41.1), and the median delay since initiation of the first second-line treatment and last follow-up was 5 years (0–23.8). At the last follow-up, 115/141 patients (74%) were in CCR (*n* = 41) or CR (*n* = 64) for both ITP and AIHA. In the subgroup of children with a favorable outcome, and who had received only first-line therapies, 39/48 children (81%) were in CR or CCR at the last follow-up. Finally, 5-year ITP and AIHA RFS were 25 and 61%, respectively.

**Figure 3:**
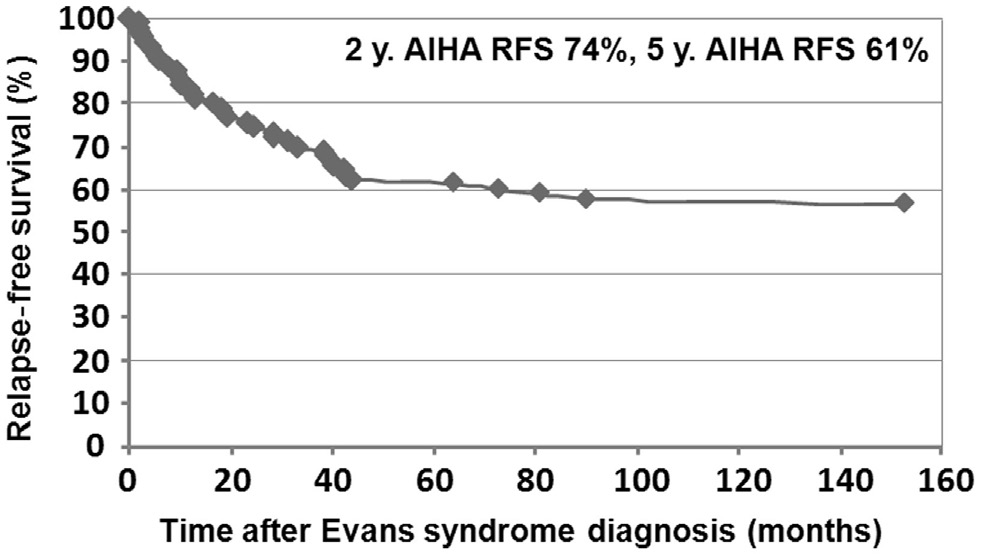
**Kaplan–Meier AIHA relapse-free survival in 90 children**.

**Figure 4:**
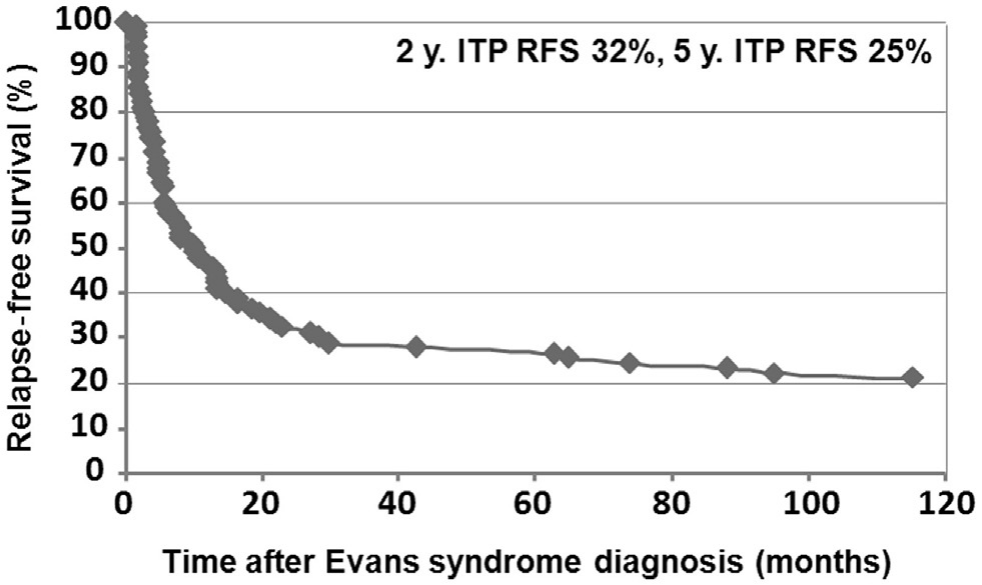
**Kaplan–Meier ITP relapse-free survival in 90 children**.

Relapse-free survival analyses were conducted in the first 90 patients. Since the diagnosis of ES, 74% of the patients (67/90) had experienced a relapse, with a median delay of 8 months (41 days to 9.5 years). Among those, 52% (35/67) relapsed with ITP and AIHA, 40% (27/67) with ITP only, and 8% (5/67) with AIHA only. Kaplan–Meier RFS curves for ITP and AIHA are presented in Figures 3 and 4.

## Discussion

### Epidemiology

With the help of the French national pediatric network, the largest (to our knowledge) prospective observational study in children with ES is now available. It describes the clinical course of 156 patients reported by 26 regional centers, covering a 30-year period and spanning 1002 patient-years. Approximately 10 new pediatric ES cases are diagnosed per year according to our inclusion and exclusion criteria. Obvious biases persist due to missing records of milder cases or of PID. Some patients registered in the CEREDIH national registry for PID who had associated autoimmune bi- or tri-cytopenia were not systematically included in the OBS’CEREVANCE database (24). This incidence can now be compared to the yearly incidence of 500–800 ITP and 30–50 AIHA cases in our country (5, 11). Data collected from this prospective long-term follow-up confirm our previous observations in 36 and 99 ES patients also included in the present cohort (10, 11): a genetic familial predisposition in 25% of children, increased initial severity in half of the cases, the modest role of infections, a possible immune background in 70% of children, and the high impact of treatment complications.

### Definitions

Our data should be interpreted taking into account (1) largely but not universally accepted definitions, (2) the national common practice of performing comprehensive phenotypic and immune investigations, and (3) long-term evaluation. We have retained Evans’s and Pui’s initial definitions of the syndrome: hematological ITP and AIHA. Rare cases of combined ITP and/or AIHA and immune neutropenia registered in our cohort need to be included in this ES definition. We did not consider isolated positive DAT or antiplatelet antibodies as sufficient for ES diagnosis, unless the patients had developed authentic AIHA or ITP. In other autoimmune diseases, a strong predictive value of isolated antinuclear, antithyroid, or anti-transglutaminase antibodies regarding the onset of autoimmune disease has not always been demonstrated (25, 26). Whether these patients with autoimmune cytopenia and autoantibodies directed against other blood cells are at risk of ultimately developing a second autoimmune cytopenia still needs to be demonstrated. Since ES is characterized by considerable clinical and biological heterogeneity regarding its presentation, course and drug responsiveness, it should definitely be considered to be a “syndrome.” In a large proportion of cases, well-defined diseases such as PID or SLE may be diagnosed, even if some of them may not appear until several decades later.

### Diagnosis

Autoimmune hemolytic anemia and ES differ in children and in adults, while ITP shares common characteristics (11). In Michel et al.’s adult series including 68 ES patients, no PID was reported, and eight cases were associated with hematologic malignant conditions (2), while in our pediatric series, 3/156 PIDs were identified and no cases of cancer occurred despite significant follow-up and an adequate survey. Complete or incomplete SLE was diagnosed in 10/68 adult patients as compared to our 13/156 cases of SLE in young adults who had also developed ES when they were teenagers.

In pediatric ES, a well-defined classical etiology is currently identified in only 10% of patients at the time of ES diagnosis or later (up to 10 years follow-up), and 30% of children did not demonstrate other immune manifestation in the long run. In as many as 60% of patients, the association with other immunologic diseases suggests that ES occurs within a still-unidentified immune background. The number of genetic causes of PID is growing, which explains why we anticipate that the percentage of secondary ES will increase in the coming years.

Autoimmune cytopenias are recognized as a component of emblematic or rarer PID, where many mechanisms of autoimmunity have been proposed (3, 22, 27). Recent pediatric ES series highlighted the importance of ALPS. Autoimmune cytopenias occur frequently in this syndrome, e.g., in 47/90 (52%) ALPS patients, at any time during the course of the disease, of whom 11 had a bi- or tri-cytopenia (28). On the other hand, using an “enlarged” ES definition, Teachey et al. reported that 21/45 (47%) children and adult ES patients showed elevated DNTs and defective FAS-mediated apoptosis, and 9/45 had FAS, FASL, or CASP10 mutations. The authors claimed that ALPS may be more prevalent in childhood ES than previously thought; however, this turned out to be questionable due to inclusion and definition biases (29, 30). In our series which includes 156 non-selected ES cases, only three ALPSs were present at initial diagnosis, all exhibiting typical ALPS diagnostic criteria. In our country, children with PID are registered in the national CEREDIH registry according to accurate definitions (24), but they are not systematically included in the OBS’CEREVANCE database when they have obvious secondary autoimmune cytopenia. ES revealing or complicating ALPS or Wiskott–Aldrich syndrome may clearly be underrepresented in our cohort. With the growing number of recently identified PIDs, and refining of key phenotypic descriptions of immune manifestations, subgroups of patients will soon be identified and classified according to genetic findings.

Primary ES should definitely remain a diagnosis of exclusion (1). In the vast majority of ES cases, the combination of multiple autoimmune cytopenias may not be a simple coincidence. It is of utmost importance to actively and regularly search for an underlying abnormal immune disorder, even after the period of transition to adulthood.

### Outcome

In former series, reported mortality rates for childhood ES were 7% (3/42) (6), 30% (3/10) (9), and 36% (4/11) (8). Currently, the syndrome still has a high 10% mortality rate. In our hands, none of the deaths were due to acute anemia, and 27% (4/15) of deaths were due to bleeding: this rate in childhood remains much greater than in isolated ITP (16, 17). Of note, in an adult series including 68 patients, three patients over 60 years of age at the time of the diagnosis of AIHA died of cardiovascular complications, but none of the 16 overall deaths were of hemorrhagic origin (2). The hypothesis of autoimmune thrombopathies or specific endothelial involvement in pediatric forms can be raised, but the evidence is still missing. Besides, 8/15 (53%) of deaths were of unclear origin and were possibly related to infections in the context of an underlying disease or to intensive treatments. They occurred in young adults after a protracted course following the onset of ES. This indicates the need to prolong the duration of management by specialized physicians, and the necessity to consider preventive anti-infectious strategies, as well as to select immunosuppressive treatments according to an optimal benefit–risk ratio.

The challenges to treat ES are known since the initial publications. However, specific guidelines are still lacking and very few treatments have been licensed in children with this syndrome (4, 31). In all, 69% of our patients required one or several second-line treatments. Fortunately, 74% were finally in CR or CCR upon the last census. ITP is the most severe cytopenia. Expectant “watch and wait” strategies are here less recommended than in isolated ITP cases because of a specific risk of hemorrhage and the increased severity of associated hemolysis. We provide for the first time a panorama of the second-line treatment strategy in clinical practice. In addition to rituximab (32–34), we underline the benefits of administering classical drugs such as azathioprine, cyclosporine, or mycophenolate mofetil, whose benefit–risk ratio has rarely been assessed (35). Splenectomy is clearly less useful in ES than in isolated chronic ITP and may even be dangerous in ALPS (36). Bone marrow transplantation should be considered in severe cases with genetically identified PID. New collaborative randomized studies are required to improve the therapeutic strategies in the different subgroups of patients.

## Conclusion

Even in an apparently primary context, childhood ES should be considered as a severe disease requiring specific follow-up until adulthood. Pediatric clinicians must be aware that the risk of severe hemorrhage is greater than in classical ITP. Most patients require several second-line treatments whose burden and associated complications may be significant. The current challenges are to classify 60% of patients in well-defined subgroups and to identify the underlying immune genetic deregulations that may be helpful to determine therapeutic strategies. Regarding clinical practice, the objectives are to identify markers to predict the severity of the disease as well as the benefit–risk ratio of targeted treatments.

## Author Contributions

NA, TL, GL, and YP designed the research, participated in the clinical care of the patients, analyzed the data, and participated in writing the paper. NA, HF, and AV collected and analyzed the data. FR-L participated in the genetic diagnosis of ALPS patients. All authors were involved in the clinical care of the patients and in collecting data, critically read the manuscript, approved the final version, and agreed to be accountable for all aspects of the work.

## Conflict of Interest Statement

The authors declare that the research was conducted in the absence of any commercial or financial relationships that could be construed as a potential conflict of interest.

## Abbreviations

AIHA, autoimmune hemolytic anemia; ALPS, autoimmune lymphoproliferative syndrome; CCR, continuous complete remission; CR, complete remission; ES, Evans syndrome; ITP, immune thrombocytopenic purpura; IVIg, intravenous immunoglobulin; NR, no remission; PID, primary genetically identified immunodeficiency; PR, partial remission; SLE, systemic lupus erythematosus.
